# Robust Diagnostic Genetic Testing Using Solution Capture Enrichment and a Novel Variant-Filtering Interface

**DOI:** 10.1002/humu.22490

**Published:** 2013-12-04

**Authors:** Christopher M Watson, Laura A Crinnion, Joanne E Morgan, Sally M Harrison, Christine P Diggle, Julian Adlard, Helen A Lindsay, Nick Camm, Ruth Charlton, Eamonn Sheridan, David T Bonthron, Graham R Taylor, Ian M Carr

**Affiliations:** 1 Yorkshire Regional Genetics Service, St. James's University HospitalLeeds, LS9 7TF, United Kingdom; 2 School of Medicine, University of Leeds, St. James's University HospitalLeeds, LS9 7TF, United Kingdom; 3 Department of Pathology, University of MelbourneMelbourne, VIC 3010, Australia

**Keywords:** software, exome sequencing, sequence analysis, mutation detection

## Abstract

Targeted hybridization enrichment prior to next-generation sequencing is a widespread method for characterizing sequence variation in a research setting, and is being adopted by diagnostic laboratories. However, the number of variants identified can overwhelm clinical laboratories with strict time constraints, the final interpretation of likely pathogenicity being a particular bottleneck. To address this, we have developed an approach in which, after automatic variant calling on a standard unix pipeline, subsequent variant filtering is performed interactively, using *AgileExomeFilter* and *AgilePindelFilter* (http://dna.leeds.ac.uk/agile), tools designed for clinical scientists with standard desktop computers. To demonstrate the method's diagnostic efficacy, we tested 128 patients using (1) a targeted capture of 36 cancer-predisposing genes or (2) whole-exome capture for diagnosis of the genetically heterogeneous disorder primary ciliary dyskinesia (PCD). In the cancer cohort, complete concordance with previous diagnostic data was achieved across 793 variant genotypes. A high yield (42%) was also achieved for exome-based PCD diagnosis, underscoring the scalability of our method. Simple adjustments to the variant filtering parameters further allowed the identification of a homozygous truncating mutation in a presumptive new PCD gene, *DNAH8*. These tools should allow diagnostic laboratories to expand their testing portfolios flexibly, using a standard set of reagents and techniques.

## Introduction

Next-generation sequencing (NGS) technologies have radically transformed the approach to genetic testing, initially in academic settings but increasingly also in clinical laboratories. The massive amount of sequence data generated by a single NGS instrument can be harnessed to allow many permutations of concurrent testing of multiple genes in a number of patients. This allows a reduction in costs and a simplification of testing through the convergence of previously diverse laboratory methods. The associated innovations in data analysis have permitted the emergence of many new applications for NGS data [Shendure and Lieberman Aiden, 2012]. Clinical diagnostic laboratories in particular have been empowered to undertake increasingly ambitious sequencing projects, resulting in a greater availability and range of diagnostic tests.

The enrichment of the target DNA sequences prior to sequencing is a key step in most NGS methods, with the use of long-range PCR (LR-PCR) being a popular choice for amplifying target regions from a relatively small number of genes [Morgan et al., 2010]. With this approach, currently available diagnostic gene panels often focus on major pathological targets such as *BRCA1* (MIM #113705) and *BRCA2* (MIM #600185) for breast cancer or *MLH1* (MIM #120436), *MSH2* (MIM #609309), and *MSH6* (MIM #600678) for hereditary nonpolyposis colorectal cancer [Morgan et al., 2010].

If it is desired to screen a larger number of genes, the main choice is between a highly parallel PCR-based approach and enrichment by hybridization. Commercially available examples of the former include the access array system (Fluidigm, San Francisco, CA) and the RainDance micro-fluidics platform (RainDance Technologies, Lexington, MA), whereas alternatives such as Haloplex and SureSelect (Agilent Technologies, Santa Clara, CA) employ enrichment by hybridization. Using such reagents, diagnostic laboratories have been able to sequence increasingly broad DNA targets in larger and larger patient cohorts [O'Sullivan et al., 2012]. However, because developing and validating bespoke capture reagents can be both time-consuming and expensive, standard rather than customized commercially available enrichment reagents have increasingly come into use.

The diagnostic screening of multiple disease genes results in the identification of unprecedented numbers of sequence variants. These variants must be both identified and interpreted for likely pathogenicity before the preparation of clinical reports. If done manually, this task can be both tedious and time-consuming, resulting in significant human error. In common with many laboratories, we perform automated variant annotation using both alamut-HT (Interactive Biosoftware, Rouen, France) to annotate single-nucleotide changes or small indels and Pindel [Ye et al., 2009] for more complex variants. These steps are ideally suited to automation on dedicated servers, and typically require only infrequent configuration by an experienced bioinformatician. In contrast, the assessment of individual annotated sequence variants for possible pathogenicity requires a greater level of user input from a clinical scientist, a process that cannot easily be automated, given our current state of knowledge. This now constitutes the main bottleneck in the reporting of pathogenic variants in NGS-based diagnostic testing.

To address this issue, we have developed two programs, *AgileExomeFilter* and *AgilePindelFilter*, which are specifically designed to be used by clinical scientists on standard desktop computers. These programs permit interactive screening of variants based on their genomic location and predicted effect on protein function, as well as filtering against flexible genetic and quality criteria. To demonstrate the utility of our hybrid diagnostic pipeline, we analyzed sequence data from two patient cohorts. The first comprised 104 hereditary cancer patients, whose DNA was enriched for target sequences using a custom-designed hybridization enrichment reagent capturing the exonic regions of 36 cancer-associated genes (Supp. Table S1). The second cohort consisted of 24 individuals with a family history of primary ciliary dyskinesia (PCD), who were analyzed using whole-exome sequence data (Supp. Table S2).

## Methods

### Subjects

Subjects were recruited from the Yorkshire Regional Genetics Service at the Leeds Teaching Hospitals NHS Trust. DNA was isolated from peripheral blood leukocytes and stored in Tris–EDTA buffer (pH 8.0) using a standard salting out protocol. Cohort 1 patients (hereditary cancer) were divided into two subgroups. The first phase of the analysis was performed on 57 individuals previously screened by accredited clinical diagnostic laboratories for a subset of the genes present on the 36-gene panel. Some of these patients were described in our previous publication [Morgan et al., 2010]. Variant information was available for these positive control samples, from their corresponding clinical report. The second phase of analysis encompassed 47 patients prospectively recruited for extended hereditary cancer testing; these patients were analyzed for differing subsets of the 36-gene panel, depending on their individual family histories. The cohort comprising 24 PCD family subjects was recruited for targeted analysis of 18 already known PCD genes. Of these, 23 were affected probands, and one was the mother of an affected child. Exome-wide sequence variants were filtered according to the criteria listed in Supp. Table S3. Initially, only variants located within the 18 known PCD genes were further examined; however, if no likely pathogenic variant was identified in this way, analysis was extended to apparently autozygous regions [Carr et al., 2013]. Additionally, for five of the PCD patients in whom a mutation in the 18 known genes could not be found, a second sibling was available to aid with autozygosity mapping (three unaffected and two affected siblings).

### Capture Array Design, Library Preparation, and Sequencing

A custom-targeted hybridization capture reagent for the diagnosis of hereditary cancer was designed using the eArray system (https://earray.chem.agilent.com/earray/) (Agilent Technologies). Capture probes were targeted to the coding exons (including 20 bp of flanking sequence) of 36 known cancer susceptibility genes (Supp. Table S1). This comprised 591 coding exons and a design target of 107,683 bp. A proportion of these genes offer recognized clinical utility and these were selected for investigation in relevant patient referrals; the remaining genes were included for development purposes. Samples from the PCD cohort were sequenced following hybridization capture using the Agilent V5 exome reagent. Genomic DNA (3 μg) was first sheared into 200–300 bp fragments using a Covaris S2 (Covaris, Inc., Woburn, MA) and then purified using a QiaQuick column (Qiagen, Chatsworth, CA). Illumina-compatible sequencing libraries were prepared using Agilent library preparation reagents as per the manufacturer's instructions. A five-cycle enrichment PCR was used to generate the libraries, which were captured using the relevant hybridization probe set. Either 16 or 10 cycles of posthybridization enrichment PCR were performed, for the cancer gene capture reagent or the exome reagent, respectively. During the first phase of the hereditary cancer study, 10 indexed libraries were pooled and sequenced on one lane of an Illumina HiSeq 2000 (Illumina, Inc., San Diego, CA). Following initial analysis, the number of pooled libraries was increased to 12 for the second phase. Also, following the first phase of the hereditary cancer study, protocol recommendations were updated to enable PCR amplification directly from the Dynabeads® (Life Technologies Ltd., Paisley, UK). Sequencing of the PCD cases was performed using both a HiSeq 2000 and HiSeq 2500 in high output and rapid modes, respectively (see Supp. Table S4 for details). Four to six cases were pooled per lane, depending on the run configuration. In all cases, paired-end 100-bp reads were generated.

### High-Throughout Informatics Pipeline (Fig.1A)

Raw data were converted to fastq.gz format and demultiplexed using CASAVA v.1.8.2 (Illumina, Inc.). The per-patient sequence data were aligned to the human reference genome (hg19) using bwa v.0.6.2 [Li and Durbin, 2009]. Sam file processing and duplicate removal were performed using Picard v.1.85 (http://picard.sourceforge.net). GATKLite v.2.3-4 was used to carry out indel realignment, base quality score recalibration, variant discovery, and read depth analysis on the duplicate-removed coordinate-sorted bam files [DePristo et al., 2011]. UnifiedGenotyper-created VCF files were annotated with positional and functional information for each variant using alamut-HT v.1.1.5 (http://www.interactive-biosoftware.com/software/alamut/overview). Default parameters were selected for all programs except the GATK UnifiedGenotyper. For this tool, both SNPs and indels were identified concurrently and the argument -minIndelFrac was adjusted to 0.15. A variant pathogenicity score was appended to variants that had been previously identified by the Yorkshire Regional Genetics Service using a curated variant list.

**Figure 1:**
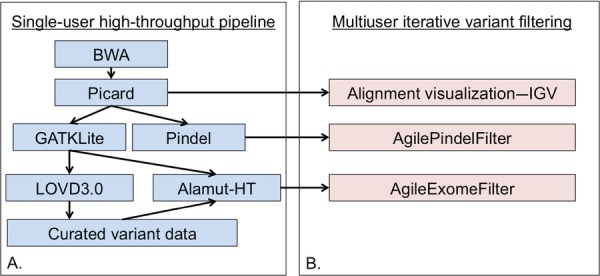
Informatics pipeline for the analysis of targeted capture data. A: The high-throughput pipeline will typically be run centrally, with dissemination of the processed data to diagnostic end-users (B) for further filtering and interpretation.

The alamut-HT output formed the basis of a variant report that could be filtered using a range of parameters with *AgileExomeFilter* (Fig.1B; http://dna.leeds.ac.uk/agile). To archive the variant data and calculate variant frequency information, VCF files outputted by the UnifiedGenotyper were imported into a local instance of LOVD3.0 [Fokkema et al., 2011]. Variant information was periodically downloaded from this database, allowing the number of observations of each point mutation to be appended to alamut-HT reports as appropriate. All reported variants have been uploaded to a LOVD database accessible at http://databases.lovd.nl/shared. BWA-aligned bam files were screened using Pindel v.0.2.4t to identify insertion/deletion variants [Ye et al., 2009]. Data output from Pindel was analyzed using *AgilePindelFilter* (http://dna.leeds.ac.uk/agile). Manual inspection of the aligned reads was performed using the Integrative Genomics Viewer v.2.2.13 [Thorvaldsdóttir et al., 2013].

### Concurrent Testing Using Locally Available LR-PCR NGS Assays

The Yorkshire Regional DNA Laboratory sequenced a proportion of the 47 phase 2 hereditary cancer samples using their standard LR-PCR NGS diagnostic assays. This included screening of 28 patients for *BRCA1* and BRCA2; 24 for *MLH1*, *MSH2*, and *MSH6*; 20 for *TP53*; and 13 for *APC* and *MUTYH*. LR-PCR amplicons encompassed the coding regions and 20 bp of flanking sequence of each target gene. The LR-PCR NGS workflow followed was similar to that described in Morgan et al. (2010), with improvements including library preparation on the Beckmann Coulter SPRIworks robot and 150-bp paired-end sequencing on an Illumina MiSeq. Variant analysis of samples tested via LR-PCR was performed using NextGENe v.2.14 (http://www.softgenetics.com/NextGENe.html) (SoftGenetics, PA).

### Variant Confirmation

Variants identified via NGS and included on a clinical report were confirmed by Sanger sequencing using an ABI3730 (Applied Biosystems, Paisley, UK). Sequence chromatograms were scored using Mutation Surveyor v.3.2 (http://www.softgenetics.com/mutationSurveyor.html) (SoftGenetics).

### SNP Analysis

In five PCD pedigrees for which no pathogenic variant was identified within the 18 known PCD genes, autozygous candidate regions were identified. Genotypes of index cases and unaffected (three pedigrees) or affected (two pedigrees) siblings were determined using the Affymetrix SNP 6.0 platform, and the data were analyzed using AutoSNPa to identify regions of homozygosity concordant between affected siblings [Carr et al., 2006].

## Results

### Sequencing and Alignment Metrics for Capture-Enriched Sequencing Libraries

Sequencing was performed on an Illumina HiSeq in both high output and rapid modes (run time of 11 days or ∼27 hr, respectively). Rapid mode runs are ideally suited to clinical testing, since fewer samples are required to initiate a sequencing batch and cluster generation can be performed on-board the HiSeq instrument, obviating the need to use a cBot. We obtained a remarkably even per-sample read distribution within each pool of sequenced libraries (Supp. Fig. S1). The range of percentages of total reads was 21.5%–29.7% for a four-plex, 11.8%–19.5% for a six-plex, 8.6%–13.3% for a 10-plex, and 6.0%–10.4% for a 12-plex. The number of pooled hereditary cancer samples was increased from 10 to 12 per lane (the maximum number of available indexes) following preliminary coverage analysis of the first phase of samples. After sample demultiplexing, the mean number of reads was therefore 41.8 million and 33.4 million for phase 1 and phase 2, respectively.

More than 99% of raw sequence reads were aligned to the hg19 reference sequence for all hereditary cancer samples. A large proportion of these reads were flagged as PCR duplicates (Supp. Fig. S2). The highest duplicate rate was identified in the phase 1 cancer cohort (mean = 79.9%, range = 65.8%–91.2%). A number of process improvements, notably performing an on-bead posthybridization PCR, may have contributed to improving the PCR duplicate rate for phase 2 (mean = 54.3%, range = 37.8%–69.9%). Nonetheless, the small 36-gene capture region makes it inevitable that a significant proportion of reads identified as PCR duplicates are actually genuinely coincident independent replicates, which are therefore being discarded unnecessarily. Theoretically, therefore, the number of samples that could be pooled per lane could thus be considerably increased. Following duplicate removal, a mean of 8.2 million and 14.5 million aligned reads remained for phase 1 and phase 2 cancer samples, respectively.

For exome analysis, the mean number of reads per patient was 114 million. As expected, the duplicate rate was considerably lower (11.1%), reflecting the much larger target size and consequent paucity of truly coincident independent reads.

### Coverage of Target Regions

Within the range of read numbers generated in these experiments, the number of reads mapping to target regions scaled linearly with the total number of aligned, duplicate-removed reads (Supp. Fig. S3), Thus, if the number of reads needed to achieve 100% variant detection sensitivity using the informatics pipeline were defined, a maximum number of samples that could be pooled per sequencing lane could be derived. In practice, the number of available sequence indexes with which the patients could be “barcoded” would probably impose limits.

Opinion varies concerning the read depth statistics required for reliable variant calling. For diagnostic use, we currently aim for 50-fold coverage before accepting the genotype at a given genomic position. By this standard, all positions in 587 of the 591 targeted exons (plus 20 bp flanking sequence) could be genotyped in the hereditary cancer cohort. Mean sequencing depth per base for phase 1 samples was 1,999, rising to 4,035 for phase 2 samples. For four targets, coverage dropped below 50-fold, one within the *ATM* gene and three within *CDKN2A* (Supp. Table S5). For the *ATM* exon and one of the *CDKN2A* exons, this occurred in one patient only, whereas the other two *CDKN2A* exons displayed reduced coverage across multiple patients.

Approximately 90% of target regions in the 18 analyzed PCD genes were covered to a depth of at least 30 (Supp. Table S6). All PCD patients in this study were consanguineous, and since homozygous variants can be identified at lower read depths than heterozygous ones, it might be acceptable to pool more patient samples in this situation. Our present methodology succeeded in identifying the heterozygous pathological variant in an unaffected carrier mother. The reduced read depth of exome-based NGS will undoubtedly reduce sensitivity of detection of some individual variants. However, this is offset by a considerably lower cost than that of analyzing 18 genes by non-NGS methods. A corollary is that two goals of diagnostic NGS sequencing should be the coherent reporting of the regions for which low coverage was identified and defining the mutation detection sensitivity in genes with known hot spots.

### Performance of the Automated Pipeline

High-throughput multistep computational pipelines for the analysis of genomic data have been employed for many years at large genome centers. Their benefits include automation with limited user interaction and an ability to finely tune the analytical parameters to each laboratory's specific requirements. All individual components of such pipelines must be known to work separately and in tandem to ensure data preservation en route to the final output. We validated our local pipeline running on a unix server with a large attached storage array (Fig.1A) by reanalysis of the phase 1 inherited cancer cohort. An average of 82 variants across the 36 genes was identified in each phase 1 patient. A subset of these was known from previous diagnostic testing (depending on the nature of the original referral request). The number of unique variants previously identified in this cohort was 116, comprising 92 transition/transversion variants and 24 indels. Of particular note was the ability of our NGS pipeline to detect a 31-bp *MLH1* deletion and a 40-bp *BRCA1* deletion (Table1). One error in the LR-PCR was highlighted, due to a deletion of *MLH1* exons 16–19. This deletion removed a LR-PCR primer binding site. The *MLH1* variant (NM_000249.3) c.1668-19A>G present on the nondeleted allele was consequently called as homozygous in the LR-PCR assay. With capture enrichment, this position was correctly scored heterozygous, since the nearby deletion event did not interfere with the capture assay. Taking these data into account, the total number of concordant genotypes across these samples was 313. Capture NGS results were 100% concordant with genotypes known from previous analysis.

**Table 1:** A Summary of Known Deletion and Insertion Variants in the Phase 1 Hereditary Cancer Samples

Gene	Transcript	Variant	Codon change	I/D	Number affected bases
*BRCA1*	NM_007294.3	c.2475del	p.Asp825Glufs*21	D	1
*BRCA1*	NM_007294.3	c.3005del	p.Asn1002Thrfs*22	D	1
*BRCA1*	NM_007294.3	c.4806del	p.Gln1604Asnfs*2	D	1
*MLH1*	NM_000249.3	c.207+1del		D	1
*MLH1*	NM_000249.3	c.1489del	p.Arg497Glyfs*11	D	1
*BRCA2*	NM_000059.3	c.3545_3546del	p.Phe1182*	D	2
*BRCA2*	NM_000059.3	c.6275_6276del	p.Leu2092Profs*7	D	2
*BRCA2*	NM_000059.3	c.7673_7674del	p.Glu2558Valfs*7	D	2
*MSH2*	NM_000251.1	c.1226_1227del	p.Gln409Argfs*7	D	2
*MLH1*	NM_000249.3	c.1744_1746del	p.Leu582del	D	3
*MSH2*	NM_000251.1	c.1786_1788del	p.Asn596del	D	3
*APC*	NM_001127510.2	c.1875_1878del	p.Asn627Leufs*2	D	4
*BRCA2*	NM_000059.3	c.6944_6947del	p.Ile2315Lysfs*12	D	4
*FLCN*	NM_144997.5	c.890_893del	p.Glu297Alafs*25	D	4
*MSH2*	NM_000251.1	c.1457_1460del	p.Asn486Thrfs*10	D	4
*PTEN*	NM_000314.4	c.956_959del	p.Thr319Lysfs*24	D	4
*APC*	NM_001127510.2	c.3183_3187del	p.Gln1062*	D	5
*MSH2*	NM_000251.1	c.2502_2508del	p.Asn835Leufs*4	D	7
*BRCA2*	NM_000059.3	c.8736_8744del	p.Asp2913_Ala2915del	D	9
*MLH1*	NM_000249.3	c.197_207+20del	p.Thr66Lysfs*9	D	31
*BRCA1*	NM_007294.3	c.1175_1214del	p.Leu392Glnfs*5	D	40
*FLCN*	NM_144997.5	c.347dup	p.Leu117Alafs*16	I	1
*MSH6*	NM_000179.2	c.4001+4_4001+8dup		I	5
*BRCA2*	NM_000059.3	c.7762_7764delinsTT	p.Ile2588Phefs*60	D/I	3

I, insertion variant; D, deletion variant.

Despite the apparently robust performance of this automated pipeline, the resulting large variant datasets require interpretation and downstream analysis from individuals with experience and knowledge of the molecular basis for each disease tested. Although the annotated data files exported by alamut-HT and Pindel can be further analyzed by eyes, their size makes this laborious and error prone. Consequently, we created two novel programs, *AgileExomeFilter* and *AgilePindelFilter* (Fig.1B) to assist with this.

### Interactive Variant Filtering Using *AgileExomeFilter*

**Figure 2:**
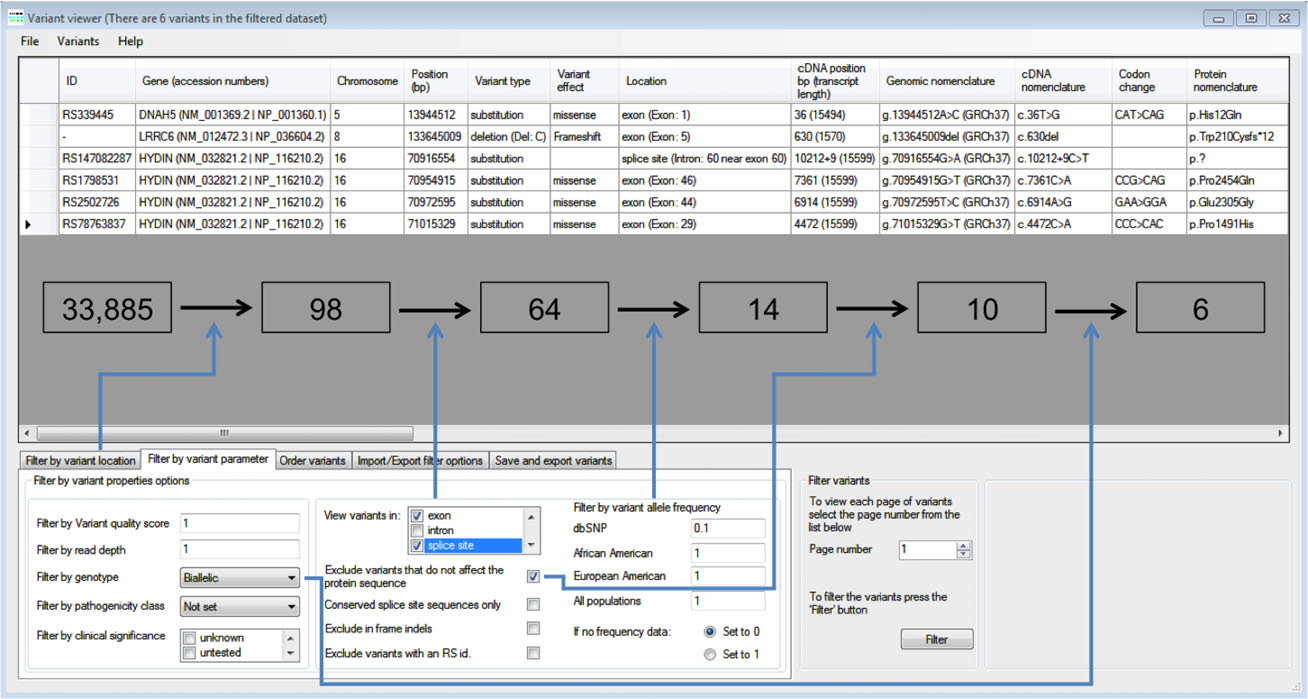
The *AgileExomeFilter* user interface. Variants are displayed in rows, with the column headers outlining the annotation information for each variant. The lower panel allows selection of filtering criteria such as zygosity status, variant location, and allele frequency. Boxed numbers indicate the reduction in variant count following the selected filtering criteria (blue arrows), which lead to the identification of the pathogenic homozygous *LRRC6* mutation (NM_012472.3) c.630del (p.Trp210Cysfs*12).

*AgileExomeFilter* allows the rapid sifting of variants annotated by alamut-HT, with each variant that passes the active filters being displayed as a single row in a grid (Fig.2). (To ease visualization, some alamut-HT data fields are combined or omitted.) To reduce false positives, variants can be filtered on their VCF quality score and their read depth. Remaining variants can then be screened using flexible user-specified criteria such as their location within a set of specified genomic locations and/or within a set of specified disease genes. If the mode of inheritance is known, variants can be filtered by zygosity, for example, (1) genes with homozygous or possibly compound heterozygous variants, for recessive disorders; (2) homozygous variants only (recessive inheritance with parental consanguinity); and (3) heterozygous variants only (dominant inheritance). Finally, variants can be filtered according to their likely effect on gene product function. These parameters include selection according to position within the gene (exon, intron, or splice sites), possible effect on nearby splice sites, and possible effect of amino acid changes on protein function.

This filtering is performed in real time, and so can be iteratively adjusted to observe multiple effects on the list of candidate pathogenic variants. Conversely, to standardize the filtering of data from patients with similar phenotypes, the filtering parameters can be saved to file and reimported for subsequent analyses. (This filtering parameter file can also be used to store a log of the filtering procedure when used in a diagnostic setting.)

### Indel Viewing and Filtering Using *AgilePindelFilter*

**Figure 3:**
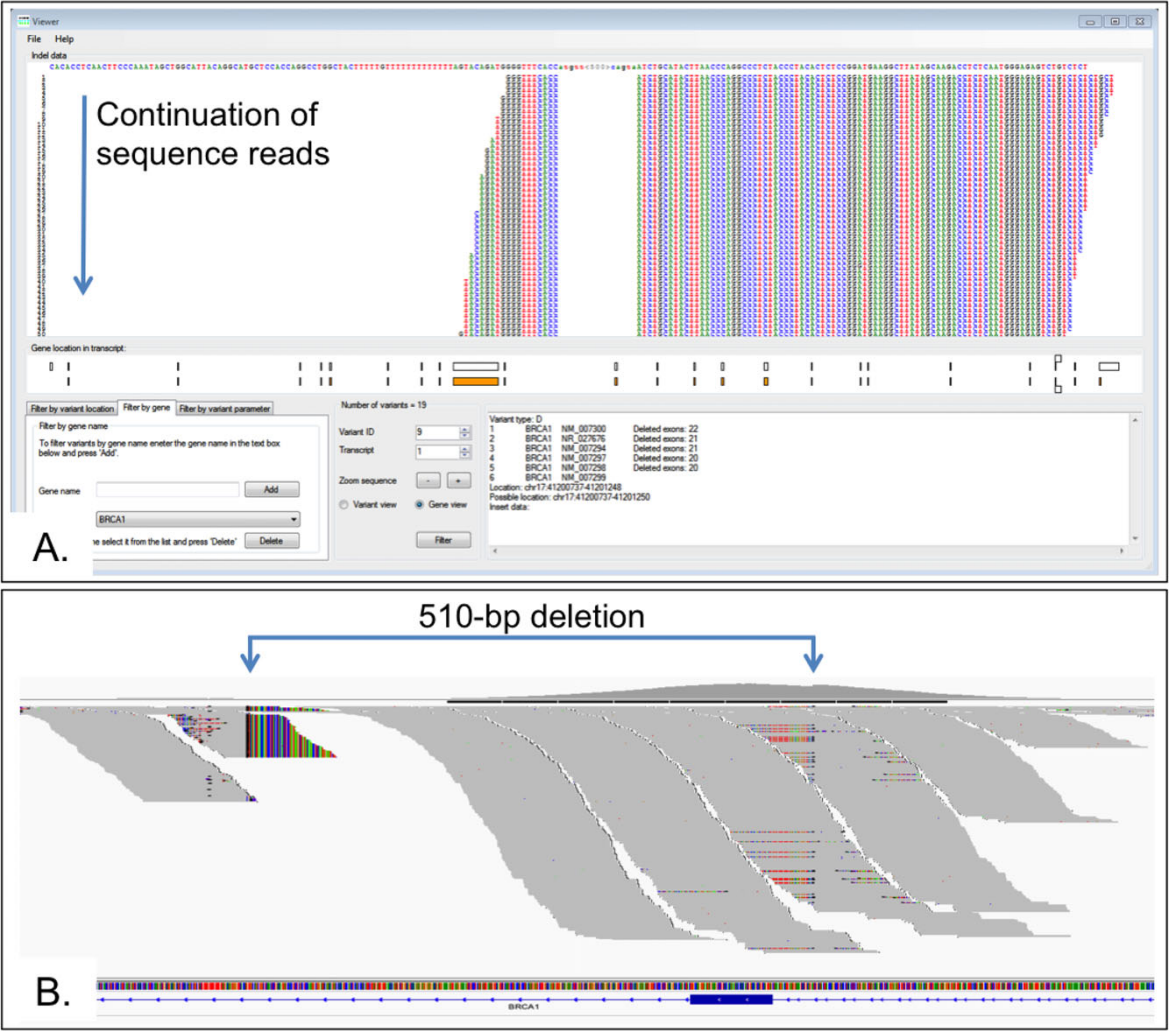
A: The *AgilePindelFilter* user interface. Reads are shown with respect to the reference sequence for a 510-bp *BRCA1* exon 22 deletion. The identified variants can be restricted to specific genes and their location within the gene. Quality parameters can be adjusted to display variants that meet a required number of reads. B: The variant shown in panel A is visible in the IGV browser by the drop in coverage and the substantial number of soft clipped reads. There is, however, no method by which the variant can be filtered out from the other sequence reads.

Because of the difficulties of identifying insertion/deletion variants within short-read NGS data, Pindel, a program specifically designed to identify indels is included in our pipeline. Pindel analyzes discordant read pairs, taking the unmapped read and performing a computationally expensive split-read alignment using a genomic search space predefined relative to the mapped read. The variants identified by Pindel can be filtered by *AgilePindelFilter* in a similar manner to the screening performed by *AgileExomeFilter*. Since Pindel exports variants in a number of different files, *AgilePindelFilter* imports from a directory containing these files. Since the Pindel variant data are more complex than that exported by alamut-HT, the data are displayed one variant at a time, with most of the user interface displaying the aligned reads relative to the reference sequence (Fig.3). Within a gene, the variant's position relative to the exon/intron structure is shown below the alignment, along with a text description of the variant.

Analysis can be restricted according to data quality, by imposing a minimum number of supported reads. This cut-off is adjustable according to the median read depth of the data and the diagnostic purpose. For example, with the inherited cancer cohort, a value of several hundred supporting reads was used, whereas the PCD exome data were limited to tens of supporting reads.

Using our automated pipeline in combination with *AgilePindelFilter*, we successfully identified all 24 insertion/deletion variants that had been previously reported in the phase one cohort (see Table1). Although these variants were also identified using the GATK variant caller's UnifiedGenotyper function, the Pindel analysis provides a second independent method that can identify insertion/deletion variants. It is recognized that searching for indels using more than one algorithm may be necessary for optimum sensitivity [Jiang et al., 2012]. Although scarce in the general population, the “difficult” indels larger than 10 bp represent an important group of disease-causing mutations.

Interestingly, Pindel detected the breakpoints of an exon 22 *BRCA1* deletion at nucleotide resolution. This variant had been previously detected using a multiplex ligation-dependent probe amplification (MLPA) assay. This deletion was captured since one breakpoint was located in the pull-down region. While reads containing the deletion could not be aligned by BWA, their paired end mates were mapped. This allowed Pindel to successful perform a split-read alignment with the reads containing the deletion and so identify this variant. This illustrates an important limitation on the detection of very large deletions, as only those with at least one end within a targeted region will be identified. Heterozygous deletions of whole exon(s) whose breakpoints are not within a capture target may still be missed unless quantitative read-count analyses prove reliable enough to permit detection.

### Clinical Validation of Hereditary Cancer Reagents

Following the successful identification of all known variants in the phase 1 cohort, we prospectively analyzed 47 further patients using the hereditary cancer panel. A mean of 84 variants per patient was identified. Diagnostic testing of an average of seven genes per patient had been requested, and for ethical reasons we restricted our analyses to the requested genes. In this way, a mean of 13 variants in five genes was identified per patient. To further maximize the validation potential of these samples, where possible, we concurrently analyzed each using our standard LR-PCR assays. With the capture reagent, we correctly identified all 480 genotypes (101 unique variants) identified by LR-PCR. Four false positive variant calls were identified within the GATK-determined variant set. These included two variants attributable to apparently misaligned reads and two variants called due to the confirmed mutation being located in the flanking sequence. No false positive insertion/deletion mutations were identified in the coding regions (±2 bp flanking intron) when concurrent analysis was performed with the LR-PCR data.

We report nine probably pathogenic variants (Supp. Table S7), of which seven would have been detected by our routine LR-PCR assay, whereas two were within genes not interrogated by the LR-PCR method. In a patient with Peutz–Jegher syndrome, a heterozygous *STK11* variant (NM_000455.4) c.566C>T (p.Thr189Ile) was identified as being probably pathogenic given previously published functional work on this variant [Karuman et al., 2001]. A further probably pathogenic heterozygous *SMAD4* variant (NM_005359.5) c.380G>T (p.Cys127Phe) was identified in a patient with multiple stomach polyps and primary cholangiocarcinoma. This variant alters a highly conserved residue in the MH1 DNA-binding domain. In all cases, variants were confirmed by Sanger sequencing.

The total number of concordant genotypes that have been tested by both the LR-PCR assay and hereditary cancer pull-down reagent is 793, comprising 147 unique variants. Based on these validation data, we consider this method to be ready and appropriate for clinical use.

### Scalability of Our Approach: Targeted Exome Analysis

A logical extension to the use of targeted capture to enrich diagnostic targets is to perform the analysis of whole exomes. We therefore applied our hybrid informatics pathway to the analysis of 24 subjects with PCD. Eighteen known genes were scrutinized in these patients, yielding a mean of 94 variants per patient. Following the filtering criteria outlined in Table2 and Supp. Table S3, we reduced the variant count to less than 15 in all cases. In nine out of 24 cases, we identified eight unique likely pathogenic variants. Of these eight variants, four have been previously reported as a cause of PCD. Subject 2010.1024 was the mother of an affected child and so was heterozygous for the identified pathogenic variant. Six variants were either nonsense or frameshift mutations, whereas the remaining two were predicted to be pathogenic by in silico analysis (Supp. Table S8).

**Table 2:** The Average Reduction in Variant Count for PCD Patients After Filtering Using *AgileExomeFilter*

Filtering parameters	Mean	Range (*n* = 24)
Total variants	33,143	31,929–34,075
Retain if located in known PCD genes	94	68–119
Retain if exonic or splice site^a^	66	49–80
Exclude if dbSNP minor allele frequency ≥0.10	15	7–21
Exclude if nonsynonymous variant	10	5–15
Retain if biallelic or homozygous	7	3–12

a Splice site is defined as 10 bp flanking the exon.

The lack of causative mutations in the remaining 15 PCD subjects might be attributable to overzealous variant filtering, but the cancer cohort experience suggested that this was unlikely to be a major factor. The lower coverage depth of the exome data might, however, result in failure to call some variants. No new candidate pathogenic variants were apparent when the filtering criteria were relaxed. As mentioned above, a subset of indel mutations probably remains undetectable without the addition of methods such as CNV detection from read depth [Shi and Majewski, 2013] or MLPA. Most likely, though, is that additional locus heterogeneity exists among the PCD cohort. To test this, we performed a simple extension to our initial 18-gene query, based on genetic criteria.

### First Example of *DNAH8* Mutation in PCD

In five families where no pathogenic variants in known PCD genes were identified, we performed SNP genotyping of the affected proband and an affected or unaffected sibling. We then searched the candidate autozygous regions in each family for homozygous deleterious variants. In one patient, a homozygous nonsense variant (NM_001371.2) c.1768C>T (p.Arg590*) was revealed in *DNAH8*. This gene encodes the ortholog of a known outer dynein arm heavy chain component in *Chlamydomonas* and on that basis has been suggested as a PCD candidate gene [Pazour et al., 2006]. However, this represents the first report of a mutation in this gene as a probable cause of PCD. Both parents were carriers of this variant and none of the three unaffected siblings were homozygous for the mutation.

## Discussion

High-throughput targeted analysis of exome data has clear diagnostic applicability to current clinical practice. We have demonstrated an unexpectedly high diagnostic rate of 42% for PCD, which exhibits high genetic heterogeneity, with many genes remaining to be identified. Particularly appealing for this type of diagnostic challenge is the fact that as new genes are identified, existing exome data can be reanalyzed without the need for additional laboratory work.

A frequent criticism is that the coverage depth of the targeted exome is significantly less than that obtainable by a smaller custom hybridization reagent, with consequent loss of sensitivity for detection of some individual mutations. Our view is that these scruples should not hinder diagnostic implementation: the increased overall diagnostic yield that accrues from exome sequencing amply justifies its use in any diagnostic setting in which the identity of the disrupted gene is open to question. From the point of view of laboratory workflow, the use of a uniform commercial reagent offers clear advantages in terms of staff time and avoids the need for revalidation of custom reagents.

Furthermore, as the cost of DNA sequencing continues to plummet, genetic testing strategies are likely to converge rapidly on one common laboratory method. This will deliver whole-genome sequence at super high coverage, eliminating current technical limitations. The remaining challenges will relate solely to data analysis and interpretation, and it is at these levels that genetic testing will need to adopt focused methods. The *Agile* software that we describe here addresses a general need for clinical scientists to be able to interrogate large variant datasets interactively, so as to make best use of their professional skills to arrive at an ultimate diagnosis. Ultimately, perhaps even this professional skill will be supplanted, but there is no indication that this will come to pass until long after whole-genome sequencing for diagnostic purposes becomes ubiquitous.

The diagnostic value of exome sequencing for genetically heterogeneous disorders has been clearly shown here, with a diagnosis rate of 42% on PCD cases. The same approach could easily be applied to other groups of rare heterogeneous diseases. The workup required to initiate such testing is minimal, involving only the scientifically informed construction of new queries using *AgileExomeFilter*.

Although we have been using NGS for clinical diagnosis of inherited disease since 2010, wider adoption of NGS as a replacement for PCR + Sanger sequencing has been slow. While some authors indicate that exome sequencing is not yet ready for the diagnostic setting, we show here that given a correctly formulated clinical question, even in genetically heterogeneous disorders high diagnostic rates can be achieved [Sikkema-Raddatz et al., 2013]. Given the ease with which *AgileExomeFilter* allows such queries to be constructed, concerns over per-variant false negative rates should not stand in the way of affordable, clinically useful exome-based diagnosis, whereas in situations where very low false negative rates are required, targeted capture reagents such as the one described here for hereditary cancer provide noticeable advantages over PCR-based methods.

## Supporting Information

Table S1. List of genes, their location and the number of exons targeted by the custom hybridization enrichment design for hereditary cancer Table S2. List of known genes, their locations and the number of target intervals screened by targeted analysis of primary ciliary dyskinesia patients Table S3. The reduction in variant count in PCD patients after filtering using *AgileExomeFilter* Table S4. The per lane run metrics for each pool of sequenced libraries Table S5. The four target regions for which coverage was <50X across both phase 1 and 2 samples Table S6. The percentage of each target interval covered to ≥30X averaged across each gene Table S7. Likely pathogenic variants identified in hereditary cancer phase 2 cases Table S8. Likely pathogenic variants identified in PCD families Figure S1. The percentage of reads sequenced per sample for each pool of sequenced libraries. Pools 1-6 are hereditary cancer phase 1 samples (n=10 samples per pool). Pools 7-10 are hereditary cancer phase 2 samples (n=12 samples per pool). Pools 11 & 12 are PCD samples (n=4 samples per pool). Pools 13-16 are PCD samples (n=6 samples per pool). Figure S2. The percentage of reads sequenced per sample that were identified as PCR duplicates for the hereditary cancer samples. Figure S3. The total number of reads mapped to the target region increases linearly with the total number of aligned reads following duplicate removal shown for the hereditary cancer samples.
