# Supplementary material for: Robust Diagnostic Genetic Testing Using Solution Capture Enrichment and a Novel Variant-Filtering Interface
Source: Hum Mutat. 2013 Dec 4;35(4):434–41. doi: 10.1002/humu.22490 (PMC4285299; doi:10.1002/humu.22490)
Supplement: Supplementary file 1 — Table S1. List of genes, their location and the number of exons targeted by the custom hybridization enrichment design for hereditary cancer Table S2. List of known genes, their locations and the number of target intervals screened by targeted analysis of primary ciliary dyskinesia patients Table S3. The reduction in variant count in PCD patients after filtering using AgileExomeFilter Table S4. The per lane run metrics for each pool of sequenced libraries Table S5. The four target regions for which coverage was <50X across both phase 1 and 2 samples Table S6. The percentage of each target interval covered to ≥30X averaged across each gene Table S7. Likely pathogenic variants identified in hereditary cancer phase 2 cases Table S8. Likely pathogenic variants identified in PCD families Figure S1. The percentage of reads sequenced per sample for each pool of sequenced libraries. Pools 1-6 are hereditary cancer phase 1 samples (n=10 samples per pool). Pools 7-10 are hereditary cancer phase 2 samples (n=12 samples per pool). Pools 11 & 12 are PCD samples (n=4 samples per pool). Pools 13-16 are PCD samples (n=6 samples per pool). Figure S2. The percentage of reads sequenced per sample that were identified as PCR duplicates for the hereditary cancer samples. Figure S3. The total number of reads mapped to the target region increases linearly with the total number of aligned reads following duplicate removal shown for the hereditary cancer samples. [file humu0035-0434-SD1.pdf]

Supp. Table S1. List of genes, their location and the number of exons targeted by the custom hybridization enrichment design for hereditary cancer

HGNC gene symbol	Chr	Start	Stop	Strand	Number of exons targeted	Targeted exonic sequence (bp)
MUTYH	1	45,805,926	45,794,978	-	16	1,650
FH	1	241,683,022	241,661,128	-	10	1,533
EPCAM	2	47,596,645	47,613,752	+	9	945
MSH2	2	47,630,331	47,710,088	+	16	2,805
MSH6	2	48,010,373	48,033,999	+	10	4,083
PMS1	2	190,656,536	190,742,162	+	12	2,799
BARD1	2	215,674,293	215,593,400	-	11	2,334
FANCD2	3	10,070,342	10,142,946	+	42	4,430
MLH1	3	37,035,039	37,092,144	+	19	2,271
PDGFRA	4	55,124,936	55,161,439	+	22	3,270
KIT	4	55,524,182	55,604,723	+	21	2,931
APC	5	112,090,588	112,179,823	+	15	8,532
RAD50	5	131,893,017	131,978,056	+	25	3,939
MET	7	116,339,139	116,436,178	+	20	4,227
NBN	8	90,996,789	90,947,810	-	16	2,265
CDKN2A	9	21,994,330	21,968,228	-	5	912
BMPR1A	10	88,635,776	88,683,476	+	11	1,599
PTEN	10	89,624,227	89,725,229	+	9	1,212
MRE11A	11	94,225,967	941,53,291	-	19	2,127
ATM	11	108,098,352	108,236,235	+	62	9,171
CDK4	12	58,145,500	58,142,308	-	7	912
BRCA2	13	32,890,598	32,972,907	+	26	10,257
RAD51	15	40,990,957	41,023,376	+	9	1,020
BLM	15	91,290,623	91,358,509	+	21	4,254
PALB2	16	23,652,478	23,614,780	-	13	3,561
CDH1	16	68,771,319	68,867,402	+	16	2,649
TP53	17	7,579,912	7,572,927	-	12	1,263
FLCN	17	17,131,451	17,116,969	-	11	1,898
RAD51D	17	33,446,632	33,427,972	-	11	1,166
BRCA1	17	41,276,113	41,197,695	-	23	5,658
RAD51C	17	56,770,005	56,811,583	+	9	1,135
BRIP1	17	59,938,900	59,760,657	-	19	3,750
AXIN2	17	63,554,738	63,526,094	-	10	2,532
SMAD4	18	48,573,417	48,604,837	+	11	1,659
STK11	19	1,206,913	1,226,646	+	9	1,302
CHEK2	22	29,130,709	29,083,885	-	14	1,632
Total					**591**	**107,683**

Start denotes the genomic position of the first nucleotide in the first targeted exon. Stop denotes the genomic position of the last nucleotide in the final targeted exon. All co-ordinates are relative to the hg19 human reference genome. Chr = chromosome.

Supp. Table S2. List of known genes, their locations and the number of target intervals screened by targeted analysis of primary ciliary dyskinesia patients

HGNC gene symbol	Chr	Start	Stop	Strand	Number of target intervals	Targeted sequence* (bp)
CCDC39	3	180,397,168	180,332,709	-	21	3,854
DNAH5	5	13,944,547	13,692,093	-	79	17,035
RSPH9	6	43,612,836	43,638,724	+	6	1,206
RSPH4A	6	116,937,787	116,953,604	+	6	2,391
HEATR2	7	769,300	825,290	+	12	2,555
DNAH11	7	21,582,864	21,940,872	+	82	16,853
NME8	7	37,889,869	379,36,694	+	15	2,367
LRRC6	8	133,687,739	133,584,554	-	12	1,885
DNAI1	9	34,459,004	34,520,754	+	21	3,186
DNAL1	14	74,111,743	74,162,645	+	8	893
DNAAF2	14	50,101,867	50,100,005	-	3	2,634
HYDIN	16	70,841,483	70,841,483	-	86	18,952
DNAAF1	16	84,179,046	84,211,447	+	12	2,706
CCDC103	17	42,978,367	42,980,185	+	3	849
DNAI2	17	72,277,957	72,310,355	+	13	2,683
CCDC40	17	78,010,462	78,064,198	+	22	4,715
CCDC114	19	48,822,028	48,800,233	-	12	2,609
DNAAF3	19	55,678,016	55,670,430	-	12	2,386
Total					**425**	**89,759**

Start denotes the genomic position of the first nucleotide in the first targeted exon. Stop denotes the genomic position of the last nucleotide in the final targeted exon. All coordinates are relative to the hg19 human reference genome. Chr = chromosome. *Exonic sequence plus 20 bp flanking region, overlapping intervals are merged into one target interval.

Supp. Table S3. The reduction in variant count in PCD patients after filtering using *AgileExomeFilter*

Filtering parameters	2001.2824	2008.2190	2009.3066	2010.0871	2010.1024	2010.3902	1997.1190	2009.3070	2009.4363	2009.4772	2009.4963	2009.5819	2011.6836	2003.0629	12.08641	12.07095	12.06153	2011.3154	2010.3971	12.05476	12.06507	12.07268	12.07319	2009.5735
**Total variants**	33, 885	32, 532	32, 904	32, 152	33, 305	33, 823	32, 983	32, 677	33, 663	33, 708	33,5 83	33, 199	33, 626	32, 830	34, 075	32, 284	33, 659	31, 929	32, 976	33, 237	32, 812	33, 155	32, 657	33, 787
**Retain if located in target genes**	98	94	104	72	103	119	116	90	84	87	106	81	96	99	105	72	102	68	87	97	94	95	102	81
**Retain if exonic or SS**	64	65	74	52	72	78	80	61	62	62	76	59	71	67	77	50	70	49	64	67	62	70	75	63
**Exclude if dbSNP MAF ≥0.10**	14	13	16	12	14	7	13	12	20	15	19	10	16	16	15	10	17	15	16	18	15	18	21	17
**Exclude if NS variant**	10	10	9	8	10	5	8	8	12	11	13	5	12	11	10	7	12	10	11	9	10	10	15	13
**Retain if biallelic or homozygous**	6	8	9	3	8	3	4	3	10	8	12	5	10	7	10	5	6	9	9	8	8	6	12	10

SS: Splice site (defined as 10 bp flanking the exon); MAF: minor allele frequency; NS: Non-synonymous

Supp. Table S4. The per lane run metrics for each pool of sequenced libraries

Platform	Run identifier	Lane	Cohort	Pool ID	Reads PF (M)	Total Yield* (GB)	Bases Q≥30 (%)
HiSeq 2000	120914_SN7001297_0042_AD19TDACXX	1	Hereditary cancer	Pool 1	469.24	45.4	92.7
HiSeq 2000	120914_SN7001297_0042_AD19TDACXX	2	Hereditary cancer	Pool 2	475.34	45.8	92.2
HiSeq 2000	120914_SN7001297_0042_AD19TDACXX	3	Hereditary cancer	Pool 3	469.20	45.0	91.8
HiSeq 2000	120914_SN7001297_0042_AD19TDACXX	4	Hereditary cancer	Pool 4	457.30	44.2	92.6
HiSeq 2000	120914_SN7001297_0042_AD19TDACXX	5	Hereditary cancer	Pool 5	437.14	42.4	92.9
HiSeq 2000	120914_SN7001297_0042_AD19TDACXX	6	Hereditary cancer	Pool 6	374.54	36.8	94.0
HiSeq 2000	121211_SN7001297_0048_AD19UJACXX	1	Hereditary cancer	Pool 7	323.68	31.5	93.1
HiSeq 2000	121211_SN7001297_0048_AD19UJACXX	6	Hereditary cancer	Pool 8	361.10	31.5	93.5
HiSeq 2000	121221_SN7001297_0053_BD191UACXX	1	Hereditary cancer	Pool 9	471.32	43.9	89.0
HiSeq 2000	130222_SN7001297_0060_AD19MBACXX	6	Hereditary cancer	Pool 10	556.12	50.4	86.7
HiSeq 2000	130320_SN7001297_0067_BC1577ACXX	7	Primary ciliary dyskinesia	Pool 11	425.20	40.3	90.7
HiSeq 2000	130320_SN7001297_0067_BC1577ACXX	8	Primary ciliary dyskinesia	Pool 12	489.84	45.4	88.6
HiSeq 2500	130604_SN7001297_0083_AH0J97ADXX	1 & 2	Primary ciliary dyskinesia	Pool 13	553.60	40.2	69.4
HiSeq 2500	130606_SN7001297_0087_AH0JBDADXX	1 &2	Primary ciliary dyskinesia	Pool 13	318.22	31.7	95.3
HiSeq 2500	130604_SN7001297_0084_BH0TTUADXX	1 & 2	Primary ciliary dyskinesia	Pool 14	662.08	58.7	84.8
HiSeq 2500	130624_SN7001297_0092_AH0TVCADXX	1 & 2	Primary ciliary dyskinesia	Pool 15	591.92	55.1	89.0
HiSeq 2500	130624_SN7001297_0093_BH0TV6ADXX	1 & 2	Primary ciliary dyskinesia	Pool 16	600.44	56.7	90.3

Reads PF: The total number of reads passing filter (millions). *Total yield includes only those bases with a Q score ≥30. GB = GigaBases. Pool 13 was re-run due to a poor quality second read. Data was merged prior to alignment.

Supp. Table S5. The four target regions for which coverage was <50X across both phase 1 and 2 samples

Gene	Chromosome	Target co-ordinates	Number of samples for which coverage was <50X across the target	Average percentage of target covered ≥50X
CDKN2A	9	21,970,881-21,971,227	79	84.20
CDKN2A	9	21,974,456-21,974,846	1	99.50
CDKN2A	9	21,994,118-21,994,350	22	91.61
ATM	11	108,150,198-108,150,355	1	99.40

Supp. Table S6. The percentage of each target interval covered to ≥30X averaged across each gene

Gene	2001.2824	2008.2190	2009.3066	2010.0871	2010.1024	2010.3902	1997.1190	2009.3070	2009.4363	2009.4772	2009.4963	2009.5819	2011.6836	2003.0629	12.08641	12.07095	12.06153	2011.3154	2010.3971	12.05476	12.06507	12.07268	12.07319	2009.5735	Mean
CCDC39	93.9	87.3	89.0	82.7	90.4	93.4	82.1	87.8	88.1	91.3	87.5	90.8	83.6	89.1	91.9	86.8	79.0	91.8	93.3	89.6	79.5	86.0	84.8	85.7	**87.9**
DNAH5	95.7	94.5	96.2	92.0	95.3	97.0	94.0	95.7	88.6	94.7	91.6	93.8	88.6	93.0	94.1	92.7	88.7	93.5	94.5	94.2	91.2	96.0	93.8	95.6	**94.5**
RSPH9	87.6	79.9	87.7	82.6	89.8	90.0	88.2	96.6	86.6	93.9	95.6	87.9	85.2	92.5	98.7	92.5	85.4	100.0	100.0	99.6	92.5	100.0	93.7	100.0	**93.8**
RSPH4A	90.3	88.1	90.9	88.9	88.7	91.5	92.8	95.4	85.0	92.5	89.5	92.3	83.9	82.4	79.0	81.3	78.9	89.3	84.5	83.1	79.6	83.1	84.6	85.2	**86.5**
HEATR2	80.2	71.6	76.0	65.1	75.9	84.0	80.4	92.7	75.7	85.4	74.1	86.2	85.0	76.8	82.8	73.8	70.1	82.8	76.4	84.7	78.7	88.8	87.0	85.4	**79.7**
DNAH11	97.5	95.0	97.2	92.1	96.9	96.9	84.5	82.1	64.4	75.1	77.7	64.0	71.9	93.7	96.0	94.1	89.0	96.0	94.7	95.9	92.8	96.6	93.9	96.0	**95.7**
NME8	96.8	92.9	92.6	89.1	93.2	95.4	96.4	98.0	95.2	97.6	97.3	96.5	96.4	92.9	93.5	87.0	81.4	90.2	93.6	92.5	82.9	86.8	87.5	89.8	**91.6**
LRRC6	94.7	87.8	96.9	83.3	93.7	96.1	92.8	95.3	94.3	96.8	95.6	96.2	94.4	88.5	89.1	90.4	82.4	94.8	96.0	88.3	88.3	87.0	86.3	83.6	**89.1**
DNAI1	97.8	95.2	97.7	93.7	96.6	98.0	97.1	98.1	95.6	98.2	97.4	97.3	94.8	94.9	97.4	94.3	86.0	95.8	96.0	97.3	95.5	98.4	96.8	95.9	**96.3**
DNAL1	96.6	90.0	90.8	85.5	92.1	94.6	95.0	94.0	91.2	95.8	91.9	94.0	91.1	76.5	80.0	79.4	72.4	83.0	77.1	79.4	77.6	92.8	78.4	78.8	**85.0**
DNAAF2	87.6	78.4	88.5	89.9	80.8	80.0	82.1	90.7	81.7	90.1	85.3	88.6	80.4	79.4	90.0	83.8	67.7	71.7	89.5	81.1	59.6	83.3	76.7	78.8	**81.5**
HYDIN	95.3	92.6	93.4	90.9	93.3	94.8	94.1	95.0	94.6	95.8	94.6	95.0	92.6	90.8	93.4	90.9	83.1	93.2	94.0	95.1	92.4	95.6	94.4	94.3	**93.6**
DNAAF1	92.1	87.3	88.4	83.7	92.3	92.9	84.2	84.9	75.5	78.9	78.2	77.0	73.9	80.4	92.6	82.5	77.4	82.8	92.3	90.7	84.8	94.4	92.3	89.3	**89.2**
CCDC103	81.5	78.2	93.1	87.0	90.7	95.1	84.0	86.5	85.8	89.0	85.9	91.7	86.4	82.8	93.1	76.4	77.4	85.0	89.8	87.9	87.3	98.8	94.4	92.1	**89.2**
DNAI2	90.1	88.2	94.3	82.9	92.4	93.6	85.8	89.7	91.5	87.0	87.9	88.6	86.7	89.6	95.5	91.9	87.2	92.3	91.4	94.4	93.8	91.4	93.7	95.2	**92.3**
CCDC40	92.9	88.5	90.4	84.7	91.1	92.1	100.0	100.0	92.5	97.5	90.4	91.2	94.6	90.6	93.5	86.5	78.6	90.5	91.3	94.6	92.0	96.2	91.8	94.8	**91.6**
CCDC114	91.1	89.0	86.7	77.3	88.6	94.3	92.9	93.4	95.4	95.6	95.7	93.1	90.0	88.0	92.1	88.1	76.5	89.7	89.2	95.4	85.8	96.7	90.2	92.4	**90.3**
DNAAF3	76.1	64.6	69.8	61.6	75.3	77.3	96.1	95.7	89.1	92.5	90.4	91.7	85.3	74.7	74.5	64.1	48.7	76.3	78.3	78.3	77.3	84.1	74.5	77.0	**73.9**
Mean	**91.0**	**86.1**	**90.0**	**84.1**	**89.8**	**92.1**	**90.1**	**92.9**	**87.3**	**91.5**	**89.3**	**89.8**	**86.9**	**86.5**	**90.4**	**85.4**	**78.3**	**88.8**	**90.1**	**90.1**	**85.1**	**92.0**	**88.6**	**89.4**	**89.0**

Target intervals include the exonic region plus 20 bp flanking sequence.

Supp. Table S7. Likely pathogenic variants identified in hereditary cancer phase 2 cases

Sample ID	Chromosome	Position	Gene	Transcript	cNomen	pNomen	Zygosity
CC64	1	45,797,228	*MUTYH*	NM_001128425.1	c.1187G>A	p.Gly396Asp	Heterozygous
CC65	5	112,174,386	*APC*	NM_000038.5	c.3095C>G	p.Ser1032*	Heterozygous
CC66	13	32,912,352	*BRCA2*	NM_000059.3	c.3860del	p.Asn1287Ilefs*6	Heterozygous
CC67	13	32,953,577	*BRCA2*	NM_000059.3	c.8878C>T	p.Gln2960*	Heterozygous
CC68	13	32,954,030	*BRCA2*	NM_000059.3	c.9097dup	p.Thr3033Asnfs*11	Heterozygous
CC69	17	7,577,551	*TP53*	NM_000546.4	c.730G>A	p.Gly244Ser	Heterozygous
CC70	17	41,223,063	*BRCA1*	NM_007294.3	c.4868C>G	N/A (splice effect)	Heterozygous
CC71	18	48,575,186	*SMAD4*	NM_005359.5	c.380G>T	p.Cys127Phe	Heterozygous
CC72	19	1,220,473	*STK11*	NM_000455.4	c.566C>T	p.Thr189Ile	Heterozygous

Supp. Table S8. Likely pathogenic variants identified in PCD families

Sample ID	Chr	Position	Gene	Transcript	cNomen	pNomen	Zygosity	Reference
2010.1024	5	13,824,434	*DNAH5*	NM_001369.2	c.6453T>A	p.Tyr2151*	Heterozygous^	
1997.1190	6	43,612,952	*RSPH9*	NM_001193341.1	c.117C>A	p.Tyr39*	Homozygous	
2008.2190	7	825,153	*HEATR2*	NM_017802.3	c.2432-1G>C	N/A (splice effect)¶	Homozygous	
2001.2824	8	133,645,009	*LRRC6*	NM_012472.3	c.630del	p.Trp210Cysfs*12	Homozygous	Chaki et al., 2012
2010.3971	8	133,645,009	*LRRC6*	NM_012472.3	c.630del	p.Trp210Cysfs*12	Homozygous	Chaki et al., 2012
2009.4772	9	34,514,434	*DNAI1*	NM_012144.2	c.1612G>A	p.Ala538Thr§	Homozygous	Zariwala et al., 2006
2003.0629	16	84,203,918	*DNAAF1*	NM_178452.4	c.1484del	p.Pro495Glnfs*40	Homozygous	
2009.4363	17	78,023,929	*CCDC40*	NM_017950.2	c.1006C>T	p.Gln336*	Homozygous	Antony et al., 2012
2009.3066	17	78,032,755	*CCDC40*	NM_017950.2	c.1416del	p.Ile473Phefs*2	Homozygous	Antony et al., 2012

^Mother of affected individual. ¶Predicted to alter the splice site by *in silico* programs SpliceSiteFinder-like (91.3), MaxEntScan (10.9), NNSPLICE (0.9), GeneSplicer (9.9) and Human Splicing Finder (90.4). This result was verified experimentally. §PolyPhen-2 prediction = Probably damaging. The amino acid residue is highly conserved. Zariwala et al., (2006) report the *DNAI1* c.1612G>A variant in two unrelated families and indicate that it segregates in *trans* with a second deleterious variant. They further report the absence of this variant in more than 200 control chromosomes.

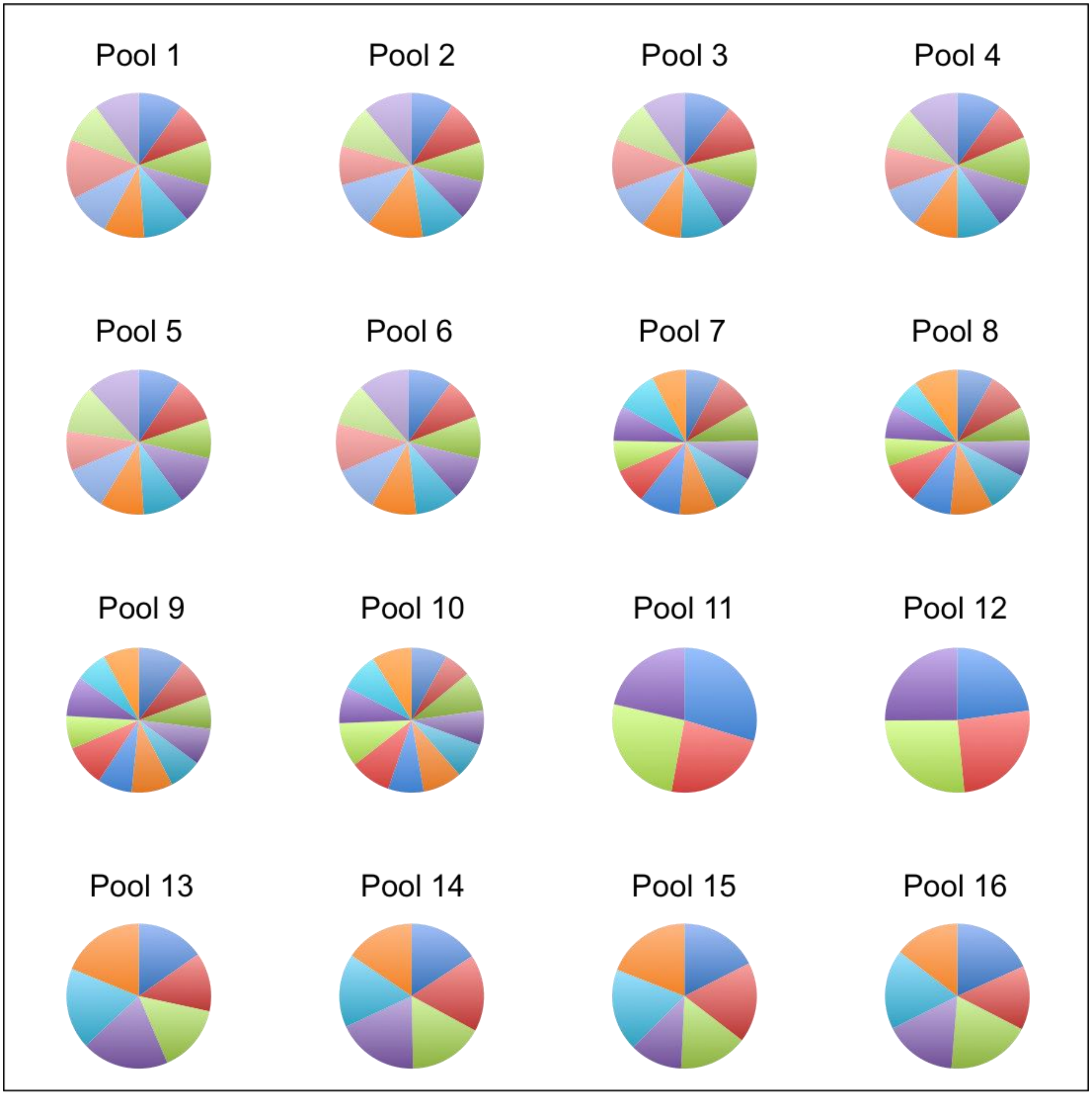

Supp. Figure S1. The percentage of reads sequenced per sample for each pool of sequenced libraries. Pools 1-6 are hereditary cancer phase 1 samples (n=10 samples per pool). Pools 7-10 are hereditary cancer phase 2 samples (n=12 samples per pool). Pools 11 & 12 are PCD samples (n=4 samples per pool). Pools 13-16 are PCD samples (n=6 samples per pool).

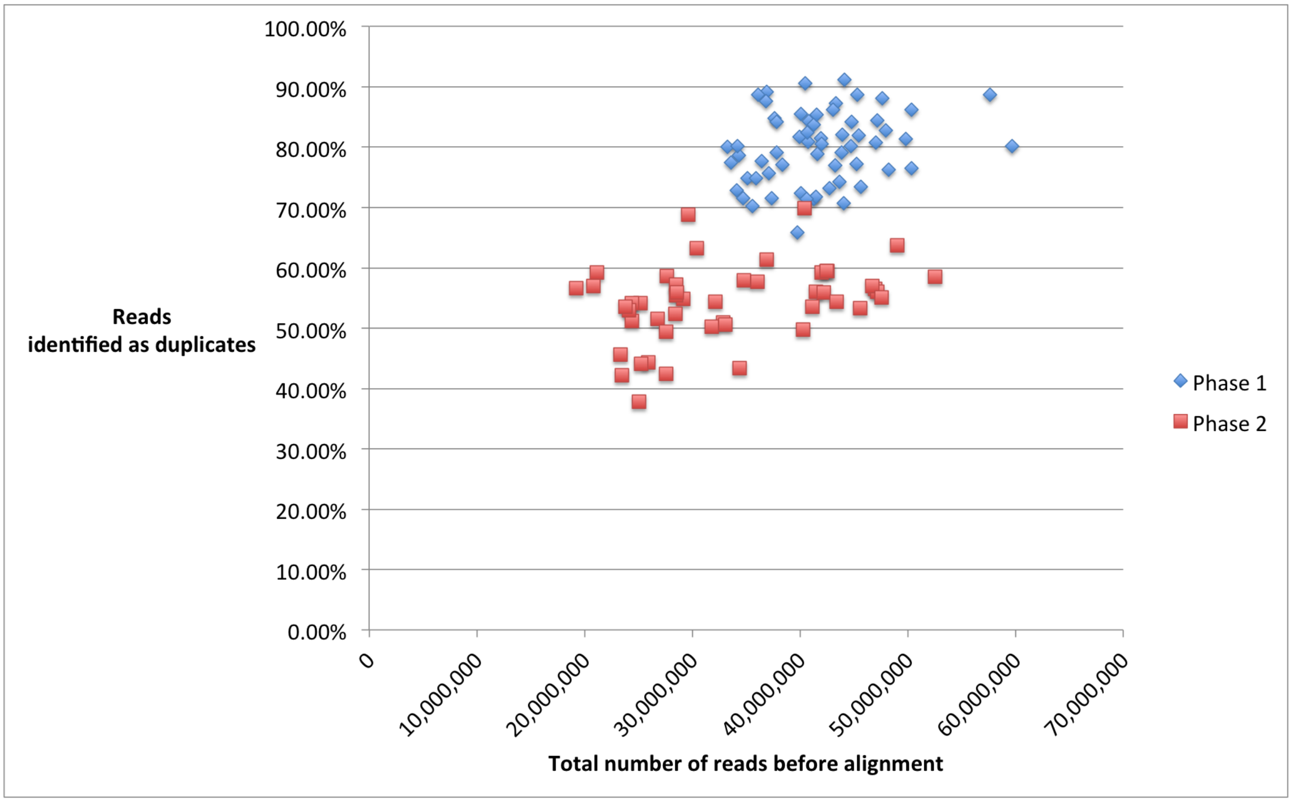

Supp. Figure S2. The percentage of reads sequenced per sample that were identified as PCR duplicates for the hereditary cancer samples.

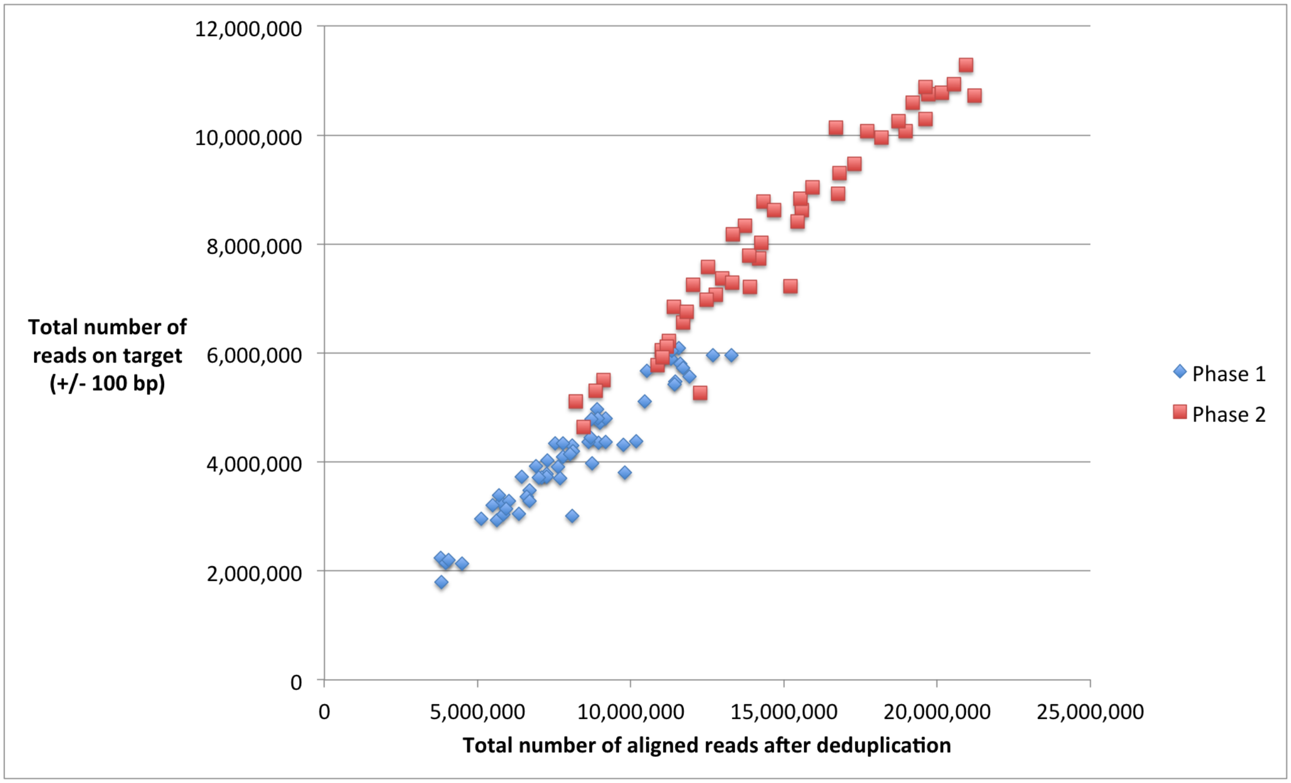

Supp. Figure S3. The total number of reads mapped to the target region increases linearly with the total number of aligned reads following duplicate removal shown for the hereditary cancer samples.
